# Biportal Endoscopic Transforaminal Lumbar Interbody Fusion for Two-Level Fusion: Hemodynamic Profiles and Early Clinical Outcomes Compared with Tubular MIS and Open Approaches

**DOI:** 10.3390/jcm15176583

**Published:** 2026-08-26

**Authors:** Tae Hoon Kang, Byungjun Kang, Geumho Lee, Jeongwoon Han, Seokin Jang, Minjoon Cho, Jung-Man Lee, Jae Hyup Lee

**Affiliations:** 1 Department of Orthopedic Surgery, Seoul National University College of Medicine, Seoul 03080, Republic of Korea; kangth1503@snu.ac.kr (T.H.K.); torto83@snu.ac.kr (M.C.); 2 Department of Orthopedic Surgery, SMG-SNU Boramae Medical Center, Seoul 07061, Republic of Korea; 3 Department of Orthopedic Surgery, Plus Orthopedic Hospital, Incheon 22009, Republic of Korea; hoishot1@naver.com; 4 Department of Orthopedic Surgery, Daniel Hospital, Bucheon 14513, Republic of Korea; eggshan91@gmail.com; 5 Department of Orthopedic Surgery, Sung-Ae Hospital, Seoul 07354, Republic of Korea; n1withetc@naver.com; 6 Spine Center, Hallym University Sacred Heart Hospital, Anyang 14068, Republic of Korea; stanley007@naver.com; 7 Department of Anesthesiology and Pain Medicine, Seoul National University College of Medicine, Seoul 03080, Republic of Korea; jungman007@gmail.com; 8 Department of Anesthesiology and Pain Medicine, SMG-SNU Boramae Medical Center, Seoul 07061, Republic of Korea

**Keywords:** spinal stenosis, spinal fusion, endoscopy, blood loss, surgical

## Abstract

**Background/Objectives**: Biportal endoscopic (BE) surgery is increasingly used for transforaminal lumbar interbody fusion (TLIF), but its hemodynamic profile and early recovery in two-level fusion remain undefined relative to tubular minimally invasive surgery (MIS) and open approaches. We compared the hemodynamic burden and early functional recovery among BE-, MIS-, and open two-level TLIF. **Methods**: In this retrospective single-center study of three sequential, non-contemporaneous cohorts, 96 patients undergoing two-level (L2–S1) TLIF (BE, *n* = 22; MIS, *n* = 39; open, *n* = 35) were analyzed. The single primary endpoint was transfusion-corrected total blood loss (corrected TBL), derived from hematocrit changes. Secondary endpoints were intraoperative estimated blood loss (EBL), drainage, allogeneic transfusion, hidden blood loss (HBL), complications, and the Numeric Rating Scale(NRS), Oswestry Disability Index (ODI), and Functional Rating Index (FRI) at 3 months. Effect sizes with 95% confidence intervals (CIs) are reported alongside *p*-values. **Results**: Corrected TBL was highest in open TLIF (2.07 ± 0.86 L) versus BE-TLIF (1.36 ± 0.54 L; mean difference −0.71 L, 95% CI −1.09 to −0.34) and MIS-TLIF (1.53 ± 0.50 L; −0.55 L, −0.88 to −0.21), whereas BE- and MIS-TLIF were comparable (−0.17 L, −0.45 to 0.12). Transfusion was required in 13.6%, 20.5%, and 60.0%, respectively (*p* < 0.001), with no difference between the minimally invasive groups. Intraoperative EBL was lower in BE-TLIF than in MIS-TLIF (−226.8 mL, −395.3 to −58.2). HBL was lowest in open TLIF (*p* = 0.030), reflecting its far larger drainage volume. At 3 months, BE-TLIF showed greater FRI improvement than MIS-TLIF (−3.97, −7.04 to −0.90); for ODI, BE-TLIF exceeded open TLIF but not MIS-TLIF. Wound problems (11.4%) and delirium (22.9%) occurred only or most often after open TLIF. **Conclusions**: In two-level fusion, BE-TLIF was associated with a lower overall hemodynamic burden and transfusion requirement than open TLIF, while corrected TBL was comparable between BE- and MIS-TLIF. Given the non-contemporaneous cohorts, baseline imbalances, limited sample size, and potential residual confounding, these findings should be considered hypothesis-generating and require confirmation in prospective or contemporaneously controlled studies.

## 1. Introduction

The prevalence of multilevel lumbar degenerative disease requiring surgical intervention is increasing with the aging population [1]. While posterior lumbar interbody fusion (PLIF) and transforaminal lumbar interbody fusion (TLIF) are standard treatments, conventional open techniques for multilevel pathology necessitate extensive subperiosteal dissection and muscle stripping. This approach-related morbidity often leads to significant blood loss, increased transfusion requirements, and prolonged postoperative pain due to paraspinal muscle atrophy [2,3].

To mitigate these issues, minimally invasive surgery (MIS) techniques such as tubular retractor-based MIS-TLIF have been widely adopted [4]. MIS-TLIF substantially reduces soft-tissue trauma and blood loss compared with open surgery. In multilevel cases, however, the restricted tubular field of view poses considerable technical challenges: the fixed working corridor limits instrument angulation and maneuverability across levels, making adequate decompression demanding, and the cumulative blood loss may still be substantial [5].

More recently, biportal endoscopic TLIF (BE-TLIF) has been introduced as a minimally invasive technique that combines endoscopic visualization with the freedom of instrument handling [6,7,8]. Prior comparative studies, including a systematic review and a meta-analysis, have reported that BE-TLIF can provide favorable hemostatic control and early pain relief in single-level surgery relative to tubular MIS or open techniques [6,7,9,10]. However, its application in two-level fusion remains controversial. Concerns persist regarding potentially prolonged operative time and irrigation-related fluid output that may mask true blood loss [9]. Moreover, few studies have directly compared the hemodynamic burden—particularly transfusion-corrected blood loss—and early functional recovery across biportal endoscopic, tubular MIS, and open approaches in a two-level cohort.

We focused on two-level fusion for two reasons. First, it is the most common multilevel construct and one in which open, tubular MIS, and biportal endoscopic approaches are all routinely used, enabling a balanced three-arm comparison. Second, at three or more levels this balance breaks down—minimally invasive techniques become disproportionately long, and midline open surgery predominates. Longer constructs extending to the sacrum also introduce spinopelvic considerations that shorter fusions do not, including acute changes in pelvic incidence [11], which fall outside the scope of the present analysis; such cases were therefore excluded to avoid heterogeneity in approach and operative burden.

The novelty of this study is threefold. To our knowledge, this is the first analysis to compare all three contemporary posterior approaches within a single two-level cohort; the first to apply transfusion-corrected, hematocrit-derived blood loss—rather than visually estimated blood loss alone—to this comparison, which is essential in a setting where more than half of the open-surgery patients received transfusion; and the first to pair that hemodynamic assessment with patient-reported functional recovery in two-level constructs.

Therefore, the purpose of this study was to compare, in two-level TLIF, the hemodynamic burden and early clinical outcomes among biportal endoscopic, tubular MIS, and open approaches. In this study, intraoperative EBL represents intraoperative hemostasis, whereas the transfusion-corrected total blood loss and the allogeneic transfusion rate together represent the overall hemodynamic burden. The single primary endpoint for statistical purposes was corrected TBL, and our primary hypothesis was that BE-TLIF would impose a lower corrected TBL than open TLIF. All other outcomes—intraoperative EBL, drainage, transfusion rate, hidden blood loss, complications, and 3-month patient-reported measures—were treated as secondary and exploratory.

## 2. Methods

### 2.1. Ethics Statement

We conducted this study in compliance with the principles of the Declaration of Helsinki. The study protocol was reviewed and approved by the Institutional Review Board of Seoul Metropolitan Government–Seoul National University Boramae Medical Center (IRB No. 40-2026-17). Owing to the retrospective nature of the study and the use of anonymized data, the requirement for informed consent was waived.

### 2.2. Study Design and Patient Selection

This was a retrospective single-center comparative cohort study, including 96 patients who underwent two-level transforaminal lumbar interbody fusion (TLIF) (L2–S1) at a single academic institution. Patients were categorized into three groups based on the surgical technique: biportal endoscopic TLIF (BE-TLIF, *n* = 22), tubular minimally invasive surgery TLIF (MIS-TLIF, *n* = 39), and open TLIF (Open-TLIF, *n* = 35).

All surgical procedures were performed by the spinal surgical team adhering to a consistent and standardized operative protocol established by the senior author. The study cohorts were defined based on the chronological evolution of surgical techniques at our institution: Open-TLIF (2011–2016), MIS-TLIF (2016–2022), and BE-TLIF (2023–2025). This sequential design reflects the natural technical evolution within the surgical team and provides a real-world comparative analysis of the primary surgical modalities employed during each respective period, tracing the transition from conventional Open-TLIF, through tubular MIS-TLIF, to the more recently adopted BE-TLIF technique [12]. Because the three cohorts were treated in non-overlapping periods, temporal confounding is an inherent concern. Perioperative care nonetheless followed the same institutional pathway throughout (see Section 2.4), and a hematocrit-based, transfusion-corrected estimate—rather than intraoperative EBL alone—served as the primary index of hemodynamic burden. Demographic and clinical variables, including age and sex (based on biological sex documented in the electronic medical records), bone mineral density, and comorbidities, were collected from medical records.

The inclusion criteria consisted of a diagnosis of degenerative spinal stenosis or spondylolisthesis requiring two-level fusion, the availability of complete perioperative laboratory data (specifically hematocrit values), and a minimum clinical follow-up of 3 months, defining the early postoperative period assessed in this study. Consistent with a previous study protocol, patients who underwent additional adjacent-level decompression procedures (e.g., unilateral laminotomy for bilateral decompression, ULBD) were also included in the analysis [9].

No a priori power calculation was performed; the sample size was determined by the number of eligible consecutive cases available during the study period. Precision was therefore lower for low-frequency outcomes such as individual complications than for the primary endpoint, and non-significant complication comparisons should not be interpreted as evidence of equivalence.

Conversely, patients were excluded if they underwent single-level or multilevel (≥3 levels) fusions, or if the procedure was a revision surgery. Patients with pathologies associated with tumor, infection, or trauma, as well as those with known coagulation disorders or anticoagulant usage that could not be safely discontinued, were also excluded. To eliminate the confounding effect of donor-site bleeding on blood loss calculations, patients who underwent autologous iliac bone grafting (AIBG) were excluded.

### 2.3. Surgical Procedures

**Figure 1:**
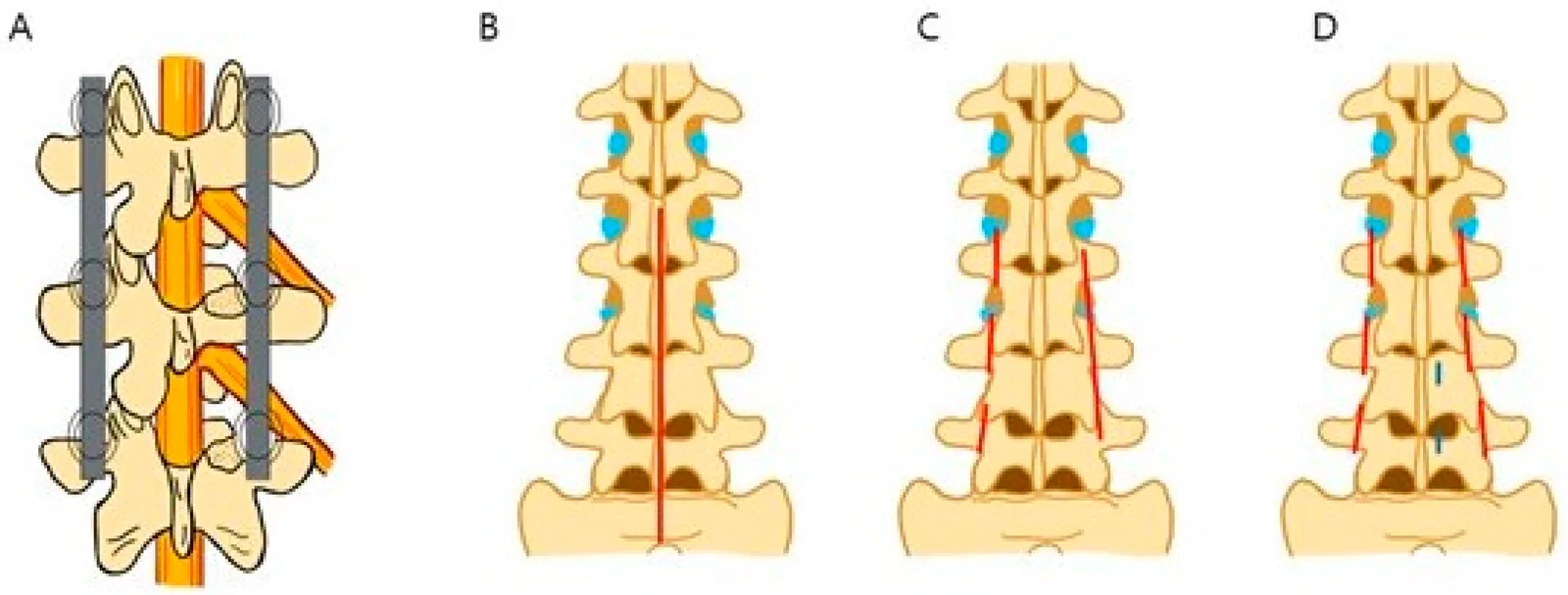
Schematic illustration of two-level TLIF (L3–L4–L5) and corresponding skin incisions for each surgical approach. (**A**) Posterior view demonstrating unilateral laminectomy for bilateral decompression (ULBD), ipsilateral facetectomies, and bilateral pedicle screw fixation in a two-level construct. (**B**–**D**) Skin incision patterns (red lines) for each approach. (**B**) Open TLIF: single long midline incision. (**C**) MIS TLIF: bilateral paramedian incisions; shorter stab incisions on the contralateral side and a longer paramedian incision on the ipsilateral side accommodate the tubular retractor. (**D**) BE-TLIF: multiple stab incisions for percutaneous pedicle screw fixations and viewing and working portals. Blue marks indicate optional accessory (quarterback) incisions for additional viewing portals or retractor placement when required.

All surgeries were performed under general anesthesia. The specific approach and hemostatic mechanisms differed among groups (Figure 1).

Open-TLIF: A standard midline incision was made, followed by extensive subperiosteal dissection of the paraspinal muscles to expose the facet joints and transverse processes bilaterally. Pedicle screws were inserted using the free-hand technique [3].

MIS-TLIF: A paramedian incision was used with a tubular retractor system. This technique preserves the muscle attachments by splitting rather than stripping the paraspinal muscles. Percutaneous pedicle screws (PPS) were placed under fluoroscopic guidance.

BE-TLIF: Two independent portals (viewing and working) were established. Continuous saline irrigation was maintained at a pressure of 30 mmHg to ensure a clear visual field and hydrostatic hemostasis. Specialized radiofrequency ablators were used for meticulous hemostasis of epidural vessels and bone surfaces. Similar to MIS-TLIF, PPS fixation was performed [8].

Despite the differences in approach, the fundamental decompression and fusion techniques were standardized across all groups. All patients underwent ULBD and ipsilateral total facetectomy to fully decompress the neural elements. Following meticulous endplate preparation to expose the bleeding bone surface, a single TLIF cage packed with local autologous bone graft obtained from the laminectomy was inserted into each intervertebral disc space. Finally, a closed suction drain was inserted subfascially in all patients to monitor postoperative bleeding and prevent hematoma formation.

### 2.4. Perioperative Management

Perioperative care followed the same institutional pathway in all three groups throughout the study period. All procedures used general anesthesia with standardized anesthetic and fluid management. Intravenous tranexamic acid had been routine institutional practice before and throughout the study period (2011–2025) and was thus applied comparably across cohorts rather than introduced only in the later minimally invasive era, although per-patient dosing was not systematically recorded. A consistent restrictive transfusion policy was maintained throughout, with allogeneic packed red blood cells given at a hemoglobin threshold of <8 g/dL, or <10 g/dL with symptomatic anemia or cardiac comorbidity. Postoperative analgesia and rehabilitation followed a standardized early-mobilization pathway, with only minor refinements over time. Discharge criteria—independent ambulation, adequate pain control on oral analgesia, and absence of wound or medical complications requiring inpatient care—were unchanged across the study period.

Intraoperative EBL was estimated by the attending anesthesiology team using a standardized method under arterial-line monitoring with serial blood gas analysis, supervised by the same senior anesthesiologist throughout the study period to limit interobserver variability across the sequential cohorts.

### 2.5. Assessment of Hemodynamic Burden and Hidden Blood Loss

To objectively quantify the hemodynamic burden, blood loss was calculated using a standardized formula based on hematocrit (Hct) changes. Serum Hct levels were measured at preoperative baseline, immediately postoperatively, and on postoperative days 1, 2, 4, and 7, following an identical schedule in all three groups.

First, the patient’s blood volume (PBV) was estimated using Nadler’s formula [13].

PBV (mL) = 0.3669 × height^3^ (m) + 0.03219 × weight (kg) + 0.6041 (for males)

PBV (mL) = 0.3561 × height^3^ (m) + 0.03308 × weight (kg) + 0.1833 (for females)

Theoretical total blood loss (TBL) was calculated using Gross’s formula at each time point [14].

TBL (mL) = PBV × (Hct_pre − Hct_post)/Hct_average

where Hct_pre is the preoperative hematocrit, Hct_post is the postoperative hematocrit, and Hct_average is the mean of the two. The maximum calculated loss among the measured time points was designated as the TBL, representing the true extent of perioperative bleeding.

Because multilevel fusion often necessitates allogeneic blood transfusion, which can mask the true decline in Hct, a transfusion-corrected calculation was applied to prevent underestimation of blood loss. One unit of packed red blood cells was estimated to contain 320 mL, consistent with the standard volume supplied by the Korean Red Cross; where the recorded transfused volume was available, that volume was used directly. Transfusions were administered postoperatively and always before the hematocrit measurement yielding the maximum calculated loss, so the correction is additive rather than duplicative. No autologous transfusion or intraoperative cell salvage was used in any group. Use of the maximum calculated loss across the postoperative series was prespecified and applied identically in all groups.

Corrected TBL (mL) = calculated TBL + transfused volume

Hidden blood loss (HBL) was then derived by subtracting the visible blood loss components from the corrected TBL [15,16]

HBL (mL) = corrected TBL − (intraoperative EBL + total drainage).

### 2.6. Assessment of Postoperative Clinical Outcomes and Complications

Postoperative recovery and clinical results were evaluated based on hospital stay, complications, and patient-reported outcome measures (PROMs). Length of hospital stay (LOS) and perioperative complications were reviewed. Specific attention was paid to postoperative delirium, dural tears, epidural hematomas, and wound problems (defined as delayed healing requiring secondary suturing or prolonged antibiotic therapy due to superficial infection). Regarding PROMs, the Numeric Rating Scale (NRS) was used for back and leg pain (0–10), while the Oswestry Disability Index (ODI) and Functional Rating Index (FRI) were used to assess functional disability. These parameters were evaluated preoperatively and at 3 months postoperatively. Clinical outcomes were analyzed based on the magnitude of change (Δ) from baseline, calculated as the postoperative minus the preoperative score; negative values therefore indicate improvement. Three-month patient-reported outcomes were available for 19 of 22 BE-TLIF patients (13.6% missing) and for all MIS-TLIF and Open-TLIF patients; analyses were performed on complete cases without imputation.

### 2.7. Statistical Analysis

All analyses were performed using Rex Pro software (v.3.6.0, based on R; Rex Software, Seoul, Republic of Korea) and Python (v3.12; SciPy v1.14). Continuous variables are presented as mean ± standard deviation with median [interquartile range], as several blood loss variables were markedly skewed; categorical variables are expressed as frequencies and percentages. Normality and homogeneity of variance were assessed with the Shapiro–Wilk and Levene tests; one-way analysis of variance was used when both assumptions held and the Kruskal–Wallis test otherwise, with Welch’s *t*-test or the Mann–Whitney U test for pairwise comparisons and Holm correction within each outcome. Categorical variables were compared using the chi-square test, or the Fisher–Freeman–Halton exact test when any expected cell count was below five, with Fisher’s exact test for pairwise comparisons. Each pairwise comparison reports the mean difference (or risk difference) with its 95% confidence interval, and Hedges’ g for continuous outcomes. Because multivariable adjustment was not feasible at this sample size, all comparisons are unadjusted and are interpreted as descriptive rather than as a test of comparative effectiveness. A *p*-value < 0.05 was considered statistically significant.

## 3. Results

### 3.1. Demographic Characteristics and Operative Data

A total of 96 patients who underwent two-level TLIF were included: 22 in the BE-TLIF group, 39 in the MIS-TLIF group, and 35 in the Open-TLIF group. The demographic and operative characteristics are summarized in Table 1. The BE-TLIF group was significantly older than the Open-TLIF group (70.3 ± 4.0 vs. 66.4 ± 7.3 years, *p* = 0.043) and comprised a higher proportion of ASA class II–III patients. The remaining baseline characteristics were comparable across the three groups.

The proportion of patients requiring additional adjacent-level decompression was significantly higher in the BE-TLIF group (40.9%) than in the MIS-TLIF (10.3%) and Open-TLIF (0%) groups (*p* < 0.001). Operative time was longer in both BE-TLIF (302.0 ± 51.9 min) and MIS-TLIF (281.4 ± 52.5 min) than in Open-TLIF (217.8 ± 30.9 min) (*p* < 0.001), with differences of +84.2 min (95% CI 59.2 to 109.2; g = 2.06) and +63.6 min (43.8 to 83.4; g = 1.44), respectively. BE- and MIS-TLIF did not differ significantly (+20.6 min, −7.4 to 48.6; *p* = 0.095), despite the fourfold higher rate of additional adjacent-level decompression in the BE-TLIF group. These baseline imbalances were not adjusted for.

### 3.2. Hemodynamic Burden and Blood Loss Parameters

The blood loss parameters are presented in Table 2. For the primary endpoint, corrected TBL was highest in Open-TLIF (2.07 ± 0.86 L) and lower in both minimally invasive groups (BE-TLIF 1.36 ± 0.54 L; MIS-TLIF 1.53 ± 0.50 L; *p* < 0.001), with large effects versus open surgery (g = −0.93 and −0.78) but no difference between BE- and MIS-TLIF (−0.17 L, 95% CI −0.45 to 0.12). Allogeneic transfusion followed the same pattern—13.6%, 20.5%, and 60.0%, respectively (*p* < 0.001)—corresponding to risk differences versus Open-TLIF of −46.4% (−68.0 to −24.7) and −39.5% (−60.1 to −18.9), and again no difference between the two minimally invasive groups (−6.9%, −26.0 to 12.3).

Intraoperative EBL was lowest in BE-TLIF (502.7 ± 288.4 mL) compared with MIS-TLIF (729.5 ± 357.5 mL; −226.8 mL, −395.3 to −58.2) and Open-TLIF (830.9 ± 598.7 mL), whereas MIS- and Open-TLIF did not differ. Total drainage was three to four times higher in Open-TLIF (1070.5 ± 404.2 mL) than in BE-TLIF (310.7 ± 134.5 mL) or MIS-TLIF (264.3 ± 170.8 mL; both *p* < 0.001; g = −2.28 and −2.62), with no difference between the two minimally invasive groups. HBL was lowest in Open-TLIF (0.17 ± 0.79 L vs. 0.55 ± 0.55 and 0.53 ± 0.51 L; *p* = 0.030) and did not differ between BE- and MIS-TLIF.

### 3.3. Postoperative Clinical Outcomes and Complications

Postoperative clinical outcomes at 3 months and perioperative complications are summarized in Table 3. LOS was substantially shorter in both minimally invasive groups than in Open-TLIF (10.7 ± 2.4 and 10.1 ± 2.5 vs. 16.6 ± 5.0 days; *p* < 0.001; g = −1.39 and −1.68), with no difference between BE- and MIS-TLIF (+0.7 days, 95% CI −0.6 to 2.0). Wound problems occurred only in the Open-TLIF group (11.4%; *p* = 0.018), which also showed the highest incidence of postoperative delirium (22.9% vs. 4.5% and 5.1%; *p* = 0.035). Incidental durotomy (4.5%, 15.4%, and 2.9%; *p* = 0.136) and symptomatic epidural hematoma (9.1%, 7.7%, and 11.4%; *p* = 0.904) did not differ. Because event counts were small, no pairwise complication comparison remained significant after Holm correction; these findings are therefore descriptive.

Patient-reported outcomes were analyzed as the change (Δ) from baseline at 3 months, available for 19 BE-TLIF, 39 MIS-TLIF, and 35 Open-TLIF patients. All three groups improved on every measure. The magnitude of improvement differed for ODI (*p* < 0.001), FRI (*p* < 0.001), and back pain NRS (*p* = 0.014), but not for leg pain NRS (*p* = 0.316). BE-TLIF showed the greatest improvement in FRI (−14.84 ± 5.27 vs. −10.87 ± 5.73 and −6.91 ± 4.69), exceeding both MIS-TLIF (−3.97, 95% CI −7.04 to −0.90; g = −0.70) and Open-TLIF (−7.93, −10.87 to −4.99; g = −1.59). For ODI and back pain NRS, BE-TLIF was superior to Open-TLIF but not to MIS-TLIF (ODI −2.67, −5.92 to 0.59; back pain −1.31, −2.65 to 0.04).

## 4. Discussion

To our knowledge, this is the first comparative analysis of blood loss profiles and early functional recovery in two-level TLIF across three surgical approaches—open, tubular MIS, and biportal endoscopic. The principal finding is that BE-TLIF was associated with a substantially lower overall hemodynamic burden—transfusion-corrected total blood loss, transfusion rate, and postoperative drainage—than open TLIF. Relative to tubular MIS-TLIF, BE-TLIF showed a lower intraoperative EBL, whereas corrected TBL, HBL, drainage, and transfusion rate were comparable between the two minimally invasive techniques; the difference between BE- and MIS-TLIF was therefore confined to intraoperative EBL, a parameter with recognized measurement limitations under continuous irrigation (discussed below). At 3 months, BE-TLIF showed the greatest improvement in the FRI, although its advantage over tubular MIS-TLIF was not evident on the ODI or on pain scores.

**Figure 2:**
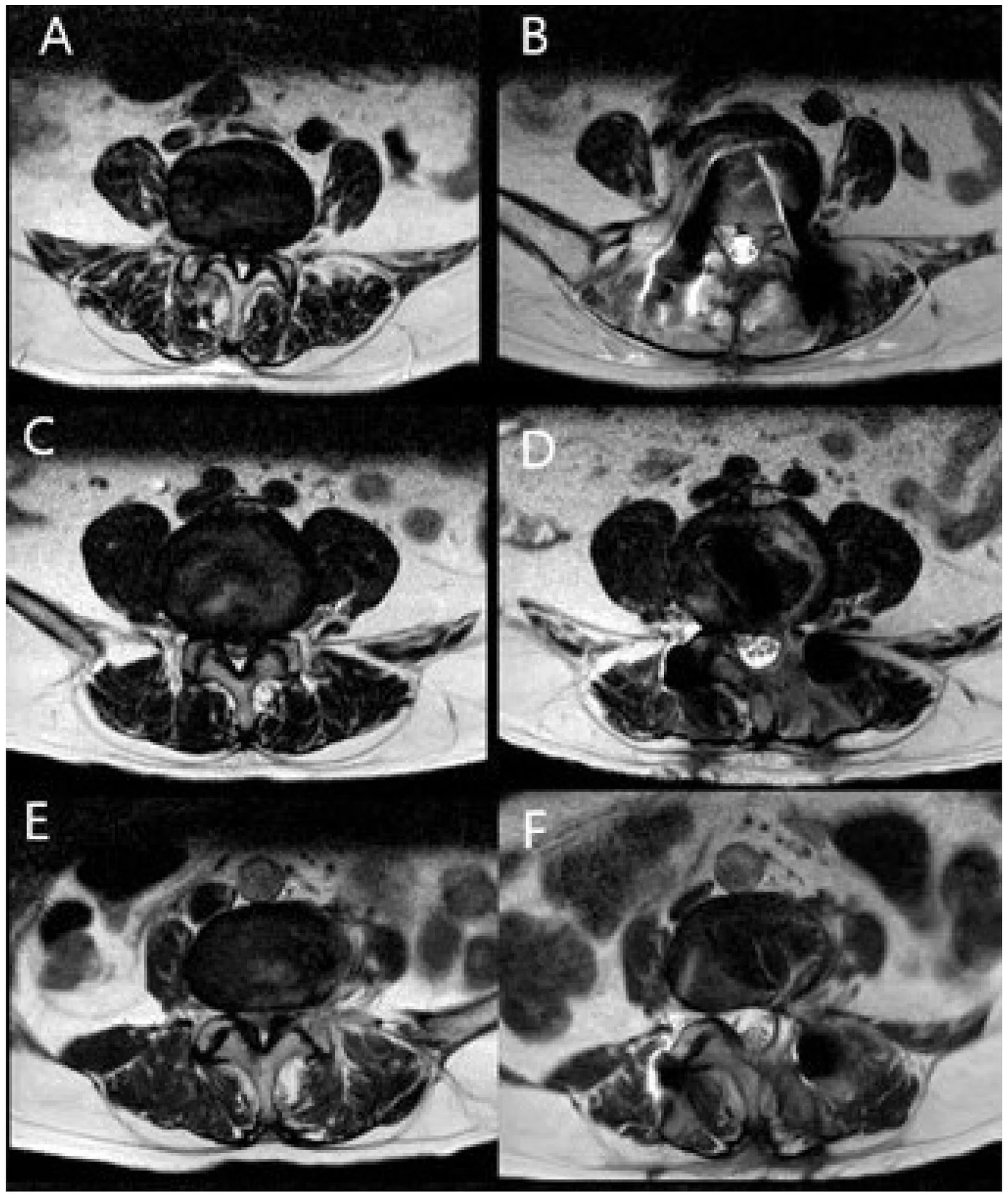
Axial MRI images demonstrating paraspinal muscle changes before and after surgery. (**A**,**C**,**E**) Preoperative MRI. (**B**,**D**,**F**) Postoperative axial MRI at 6 weeks. (**A**,**B**) Open TLIF. (**C**,**D**) MIS TLIF. (**E**,**F**) BE-TLIF. These images are illustrative examples selected from individual patients and do not represent a quantitative comparative analysis; no morphometric measurement of muscle cross-sectional area was performed, and no comparative inference regarding muscle preservation should be drawn from them.

The differences in hemodynamic burden largely reflect the extent of posterior muscle disruption inherent to each approach. Open-TLIF requires a midline incision with extensive subperiosteal stripping of the paraspinal muscles, whereas both MIS-TLIF and BE-TLIF use a muscle-sparing paramedian muscle-splitting approach with percutaneous pedicle screw fixation. The two minimally invasive techniques differ only in the working channel—a fixed tubular retractor for MIS-TLIF versus independent endoscopic portals for BE-TLIF—while the bony decompression and fusion steps (ULBD, ipsilateral total facetectomy, and interbody cage insertion) were standardized across all three groups. The intergroup differences in blood loss may therefore be associated with the surgical approach and intraoperative hemostatic environment rather than with the extent of bony resection, although a causal attribution cannot be made from this observational design. Representative axial MRI images are provided for illustration only (Figure 2); because quantitative morphometric analysis was not performed, no comparative inference regarding muscle preservation should be drawn from them.

Corrected TBL was highest in Open-TLIF and markedly lower in both minimally invasive groups, in agreement with prior open-versus-minimally invasive comparisons [2,17] and with our previous single-level analysis [9]. Between the two minimally invasive techniques, however, corrected TBL was comparable, and only intraoperative EBL was lower in BE-TLIF (503 vs. 730 mL). This isolated difference warrants caution, because EBL is the least reliable of our measures in the BE-TLIF group: under continuous saline irrigation, visual EBL estimates are biased downward by dilution and clot washout. Although EBL was estimated under arterial-line monitoring with serial blood gas analysis by a consistently supervised anesthesiology team [9,18], residual measurement error cannot be excluded. We therefore do not read the lower EBL as evidence that BE-TLIF confers superior hemostasis over tubular MIS-TLIF; corrected TBL, which was comparable between the two techniques, is the more robust index of overall hemodynamic burden. Where a genuine intraoperative difference exists, it is most plausibly explained by the endoscopic operative environment—hydrostatic irrigation and targeted radiofrequency hemostasis under magnified visualization.

Comparison with our single-level series clarifies what changes when a second level is added [9]. At one level, BE-TLIF showed both a lower intraoperative EBL and a lower total blood loss than MIS-TLIF, with no transfusions in either group, despite more frequent adjacent-level decompression in the endoscopic group—a pattern repeated in the present cohort. At two levels, calculated blood loss roughly doubled in both techniques, and BE-TLIF retained its lower intraoperative EBL; corrected total blood loss, however, no longer differed between them, and transfusion became necessary in a subset of patients in both groups. The advantage of BE-TLIF therefore appears confined to intraoperative blood loss and does not extend to the overall hemodynamic burden.

The pathophysiology of HBL is primarily attributed to blood extravasation into the dead space created during tissue dissection [19]. In this cohort, HBL was lowest in the Open-TLIF group (0.17 ± 0.79 L; *p* = 0.030), which simultaneously showed the highest total drainage (1071 mL), approximately three to four times that of the BE-TLIF and MIS-TLIF groups. Because HBL is a derived quantity—corrected TBL minus EBL and drainage—this pattern is best read as a redistribution between compartments rather than as less true bleeding: the large drainage volume after open surgery shifts blood loss from the “hidden” to the “visible” compartment.

In contrast, the MIS-TLIF and BE-TLIF techniques limit dead space by splitting muscle or applying continuous fluid pressure, resulting in minimal drainage (264–311 mL). Extravasated blood therefore remains largely within the tissues, which elevates the calculated HBL in these groups, and HBL did not differ between the two minimally invasive techniques (+0.01 L, 95% CI −0.27 to 0.30). The same derivation, however, makes HBL highly sensitive to error in its inputs: inaccuracies in EBL and drainage propagate directly into it, and the wide standard deviation in the Open-TLIF group (± 0.79 L, including negative individual values) illustrates this instability. HBL is therefore best read as a descriptive index of drainage dynamics rather than as an independent measure of tissue trauma, and corrected TBL remains the more dependable summary of overall hemodynamic burden.

A clinically important finding is the marked reduction in allogeneic blood transfusion in the BE-TLIF (13.6%) and MIS-TLIF (20.5%) groups compared with the Open-TLIF group (60.0%), corresponding to absolute risk differences of approximately 46% and 40%, respectively. Allogeneic transfusion is associated with transfusion-related immunomodulation (TRIM), a suppression of host immunity linked to an increased risk of wound complications and surgical site infection [20].

Wound problems occurred exclusively in the Open-TLIF group (11.4%), and although this is primarily attributable to the extensive soft-tissue dissection and larger incision inherent to the open approach, the higher transfusion rate may have further compounded this risk. The absence of wound problems in the BE-TLIF and MIS-TLIF groups is consistent with the combined benefit of reduced local tissue trauma and a lower systemic transfusion burden. However, with only four events in total, no pairwise comparison remained significant after correction for multiplicity, and this observation must be regarded as descriptive rather than as demonstrated superiority in safety.

Postoperative delirium followed the same pattern, occurring in 22.9% of Open-TLIF patients versus 4.5% and 5.1% of the BE- and MIS-TLIF patients, respectively (*p* = 0.035). Delirium is among the most consequential complications in elderly spine surgery, prolonging hospitalization and predicting functional decline, and it has been linked to perioperative anemia, allogeneic transfusion, and the magnitude of the surgical stress response [21]. Its distribution here therefore parallels the hemodynamic findings: the group with the highest transfusion requirement and total blood loss also had the highest incidence of delirium and the longest hospital stay. This association is clinically meaningful even though no pairwise comparison retained significance after adjustment for multiple testing, and the small number of events precludes any causal inference. Notably, the BE-TLIF cohort was the oldest of the three, so the low observed incidence is unlikely to be attributable to a younger case mix alone—although unmeasured confounding and evolving perioperative practice cannot be excluded.

Incidental durotomy was numerically less frequent after BE-TLIF (4.5%) than after tubular MIS-TLIF (15.4%), although this difference was not significant. Achieving adequate decompression through a fixed tubular retractor in multilevel surgery can be technically demanding because of restricted visualization and limited instrument angulation [5], and the magnified endoscopic view under continuous irrigation may reduce dural injury—but with eight events across 96 patients, this remains speculative.

The BE-TLIF cohort was older, had more comorbidities, and more often required additional adjacent-level decompression (40.9%) than the other groups. Despite this, BE-TLIF showed the greatest improvement in FRI at 3 months, exceeding both MIS-TLIF (−3.97, 95% CI −7.04 to −0.90) and Open-TLIF (−7.93, −10.87 to −4.99). For ODI and back pain, however, BE-TLIF was superior to open surgery but indistinguishable from MIS-TLIF, and length of stay did not differ between the two minimally invasive groups; the functional advantage over tubular MIS therefore rests on a single instrument and should not be overstated.

Although these baseline imbalances would generally be expected to disadvantage BE-TLIF, they do not exclude confounding—non-randomized technique selection, evolving indications, and unmeasured patient factors could all influence patient-reported outcomes—so the greater FRI improvement should be regarded as hypothesis-generating. Mechanistically, it may reflect the independent viewing and working portals, which allow targeted decompression of adjacent segments without extensive additional exposure, and the limited disruption of the paraspinal musculoligamentous complex [7].

This study has several limitations. First, the sequential-cohort design carries selection and temporal bias: because the techniques were used in different periods (2011–2016, 2016–2022, and 2023–2025, respectively), some differences may reflect the general evolution of perioperative care rather than the technique alone. Interbody cage design and instrumentation were not systematically recorded and are likely to have evolved over the study period; this remains an unquantifiable residual temporal confounder. Although perioperative protocols were consistent throughout, unmeasured temporal effects, confounding by indication, and the unadjusted baseline differences in age and ASA class cannot be excluded, and no contemporaneous control group was available. Our findings are therefore associational and hypothesis-generating. Second, the BE-TLIF cases were accrued from the institutional introduction of the technique in 2023 and therefore span the surgical team’s early adoption phase, whereas the comparison techniques were performed at technical maturity; we have previously characterized this learning curve in a separate analysis, and the present two-level series was accrued during and shortly after that adoption phase. Learning-curve effects would be expected to bias against BE-TLIF for operative time and complications, but their influence on blood loss and functional recovery cannot be quantified here. Third, intraoperative EBL is inherently less reliable in BE-TLIF, where continuous saline irrigation makes visual estimation difficult; we mitigated this by using hematocrit-based corrected TBL as the primary endpoint, but residual measurement error cannot be excluded, and irrigation-related hemodilution or clot washout may also influence Hct-based calculations. Because HBL is derived from these inputs, it inherits their error. Fourth, no sample-size calculation was performed, and statistical power for complications was limited, so non-significant differences in these outcomes should not be read as equivalence; the confidence intervals for the primary endpoint, however, were narrow enough to exclude clinically meaningful differences between the two minimally invasive techniques. Three-month patient-reported outcomes were missing for 3 of 22 BE-TLIF patients, and complete-case analysis may introduce bias if these data were not missing at random. Fifth, although representative axial MRI images suggest greater paraspinal muscle preservation in the minimally invasive groups (Figure 2), quantitative assessment was not performed, as postoperative MRI was unavailable for many patients in the earlier cohorts. Validated methods for assessing lumbar muscle function and fatigue are available [22], and their application in future prospective studies would allow the muscle-sparing rationale of these approaches to be tested directly rather than inferred. Sixth, although antiplatelet use did not differ significantly among groups, individual variation in hemostasis could not be fully controlled. Sex-specific factors that may influence lumbar spine pain, such as obstetric history, were also unavailable in our dataset; as most patients in this cohort were women, this remains an unmeasured source of variation in patient-reported outcomes [23]. Finally, this study addressed only early outcomes (3 months) and did not evaluate fusion rates, cage subsidence [8], or late complications, and single-center findings from one surgical team have limited external validity. Prospective studies with long-term radiographic follow-up are needed to confirm the structural durability of two-level BE-TLIF.

## 5. Conclusions

In this retrospective sequential cohort, BE-TLIF was associated with lower transfusion requirements and corrected total blood loss compared with Open-TLIF, while corrected total blood loss was comparable between BE-TLIF and MIS-TLIF. BE-TLIF was also associated with favorable early patient-reported functional outcomes, most consistently on the FRI. Given the non-contemporaneous cohorts, baseline imbalances, limited sample size, and potential residual confounding, these findings should be considered hypothesis-generating and require confirmation in prospective or contemporaneously controlled studies.

## Figures and Tables

**Table 1:** Demographic and operative characteristics.

Variable	BE-TLIF (*n* = 22)	MIS-TLIF (*n* = 39)	Open-TLIF (*n* = 35)	*p*-Value
Age (years)	70.32 ± 4.03 ^c^	69.87 ± 6.45 ^c^	66.43 ± 7.28 ^a,b^	0.043 ^†^
Sex (Male/Female)	6/16	15/24	10/25	0.562 ^§^
BMI (kg/m^2^)	26.33 ± 3.73	25.86 ± 3.26	25.72 ± 3.36	0.798 ^‡^
BMD (T-score)	−1.62 ± 0.78	−1.50 ± 1.14	−1.75 ± 0.92	0.580 ^‡^
ASA Classification(I/II/III)	0/19/3	2/32/5	12/22/1	<0.001 ^§^
Previous antiplatelet	3 (13.6%)	6 (15.4%)	9 (25.7%)	0.410 ^§^
Preop Hb (g/dL)	13.00 ± 1.38	13.35 ± 1.75	13.07 ± 1.55	0.656 ^‡^
Adj. Decom	9 (40.9%) ^b,c^	4 (10.3%) ^a^	0 (0%) ^a^	<0.001 ^§^
Op Time (min)	302.0 ± 51.9 ^c^	281.4 ± 52.5 ^c^	217.8 ± 30.9 ^a,b^	<0.001 ^†^

Values are presented as mean ± standard deviation or number of patients (%). Superscripts denote significant post hoc differences: ^a^ vs. BE-TLIF; ^b^ vs. MIS-TLIF; ^c^ vs. Open-TLIF. ^†^ Kruskal–Wallis test; ^‡^ one-way ANOVA; ^§^ chi-square test. TLIF: transforaminal lumbar interbody fusion; BE-TLIF: biportal endoscopic TLIF; MIS-TLIF: minimally invasive tubular-based TLIF; Open-TLIF: conventional open TLIF; BMI: body mass index; BMD: bone mineral density; ASA: American Society of Anesthesiologists; Hb: hemoglobin; Adj. Decom: additional adjacent-level decompression; Preop: preoperative.

**Table 2:** Hemodynamic burden and blood loss parameters.

Variable	BE-TLIF (*n* = 22)	MIS-TLIF (*n* = 39)	Open-TLIF (*n* = 35)	*p*-Value
Intraop EBL (mL)	502.7 ± 288.4 ^b,c^	729.5 ± 357.5 ^a^	830.9 ± 598.7 ^a^	0.004 ^†^
Total Drainage (mL)	310.7 ± 134.5 ^c^	264.3 ± 170.8 ^c^	1070.5 ± 404.2 ^a,b^	<0.001 ^†^
Transfusion Rate	3 (13.6%) ^c^	8 (20.5%) ^c^	21 (60.0%) ^a,b^	<0.001 ^§^
Average transfused Vol (mL)	72.7 ± 200.4 ^c^	102.6 ± 219.4 ^c^	566.9 ± 657.8 ^a,b^	<0.001 ^†^
Corrected TBL (L)	1.36 ± 0.54 ^c^	1.53 ± 0.50 ^c^	2.07 ± 0.86 ^a,b^	<0.001 ^†^
HBL (L)	0.55 ± 0.55	0.53 ± 0.51 ^c^	0.17 ± 0.79 ^b^	0.030 ^†^

Values are presented as mean ± standard deviation or number of patients (%). Superscripts denote significant post hoc differences: ^a^ vs. BE-TLIF; ^b^ vs. MIS-TLIF; ^c^ vs. Open-TLIF. ^†^ Kruskal–Wallis test; ^§^ chi-square test. TLIF: transforaminal lumbar interbody fusion; BE-TLIF: biportal endoscopic TLIF; MIS-TLIF: minimally invasive tubular-based TLIF; Open-TLIF: conventional open TLIF; Intraop: intraoperative; EBL: estimated blood loss; TBL: total blood loss; HBL: hidden blood loss.

**Table 3:** Postoperative clinical outcomes and complications at 3 months.

Variable	BE-TLIF (*n* = 22)	MIS-TLIF (*n* = 39)	Open-TLIF (*n* = 35)	*p*-Value
Length of Stay (days)	10.73 ± 2.35 ^c^	10.05 ± 2.50 ^c^	16.60 ± 4.95 ^a,b^	<0.001 ^†^
Complications, *n* (%)				
- Postop Delirium	1 (4.5%)	2 (5.1%)	8 (22.9%)	0.035
- Wound problem	0 (0%)	0 (0%)	4 (11.4%)	0.018 ^¶^
- Epidural Hematoma	2 (9.1%)	3 (7.7%)	4 (11.4%)	0.904 ^¶^
- Dural Tear	1 (4.5%)	6 (15.4%)	1 (2.9%)	0.136 ^¶^
NRS Back (Δ 3M)	−4.05 ± 2.30 ^c^	−2.74 ± 2.53	−1.80 ± 2.92 ^a^	0.017 ^†^
NRS Leg (Δ 3M)	−5.53 ± 1.68	−4.72 ± 2.25	−5.37 ± 2.40	0.316 ^†^
ODI Score (Δ 3M)	−12.95 ± 6.11 ^c^	−10.28 ± 4.70 ^c^	−6.26 ± 5.10 ^a,b^	<0.001 ^†^
FRI Score (Δ 3M)	−14.84 ± 5.27 ^b,c^	−10.87 ± 5.73 ^a,c^	−6.91 ± 4.69 ^a,b^	<0.001 ^†^

Values are presented as mean ± standard deviation or number of patients (%). Superscripts denote significant post hoc differences: ^a^ vs. BE-TLIF; ^b^ vs. MIS-TLIF; ^c^ vs. Open-TLIF. ^†^ Kruskal–Wallis test; ^¶^ Fisher–Freeman–Halton exact test. TLIF: transforaminal lumbar interbody fusion; BE-TLIF: biportal endoscopic TLIF; MIS-TLIF: minimally invasive tubular-based TLIF; Open-TLIF: conventional open TLIF; NRS: numeric rating scale; ODI: Oswestry Disability Index; FRI: Functional Rating Index; Δ: change from baseline.

## Data Availability

The data presented in this study are available on request from the corresponding author due to privacy and ethical restrictions.
